# Impact of pharmaceutical validation on prescribing errors in a neonatal intensive care unit. Randomised and controlled study

**DOI:** 10.3389/fped.2024.1346090

**Published:** 2024-04-04

**Authors:** M. D. Canales-Siguero, C. García-Muñoz, J. M. Caro-Teller, S. Piris-Borregas, S. Martín-Aragón, J. M. Ferrari-Piquero, M. T. Moral-Pumarega, C. R. Pallás-Alonso

**Affiliations:** ^1^ Department of Pharmacy, 12 de Octubre University Hospital, Madrid, Spain; ^2^ Researcher, Maternity and Childhood Health Research Group (Area 4), i+12 Research Institute, Madrid, Spain; ^3^ Department of Neonatology, 12 de Octubre University Hospital, Madrid, Spain; ^4^ Department of Pharmacology, Pharmacognosy and Botany, Complutense University, Madrid, Spain

**Keywords:** pharmaceutical validation, pre-post intervention, electronic prescription, prescription errors, neonatal intensive care unit, clinical pharmacist

## Abstract

**Purpose:**

To compare the frequency of electronic prescription errors when the prescription was validated by the clinical pharmacist vs. when it was not.

**Methods:**

This prospective randomised controlled study was conducted in three phases. A randomised phase, in which patients were divided into control and intervention groups, and a pre- and post-intervention phase were consecutively performed to analyse the impact of pharmaceutical validation of prescriptions in a neonatal intensive care unit (NICU). This study was performed at a highly complex NICU at a tertiary hospital. All patients born during the study period who were admitted to the NICU, with a stay lasting ≥24 h, and received active pharmacological treatment were included in the study. Pharmaceutical validation was performed according to the paediatric pharmaceutical care model. A high level of validation was selected for this study. In the intervention group, discrepancies found during the review process were communicated to the medical team responsible for the patients and resolved on the same day.

**Results:**

In total, 240 patients were included in this study. Sixty-two patients were allocated to the pre-intervention (*n* = 38) or post-intervention (*n* = 24) groups, and 178 patients were randomly sorted into two groups, control (*n* = 82 newborns) and intervention (*n* = 96 newborns). During the randomisation phase, the number of prescription errors detected was significantly lower in the intervention group than that in the control group (129 vs. 270; *p* < 0.001). Similarly, prescription errors reaching the patient were significantly reduced from 40% (*n* = 108) in the control group to 1.6% (*n* = 2) in the intervention group. In the pre- and post-intervention periods, the prescription lines containing prescription errors decreased from 3.4% to 1.5% (*p* = 0.005).

**Conclusions:**

This study showed that the pharmaceutical validation process decreased both the number of errors in the electronic prescribing tools and the number of prescription errors reaching the patient.

## Introduction

Reducing the rate of error throughout the course of drug administrations in inpatient care has been a key objective of the World Health Organization since 2017. The third global patient safety challenge, referred to as “Medication without harm”, was launched that year. This global initiative aimed to reduce medication errors by 50% by taking action in three priority and vulnerable areas: high-risk situations, polypharmacy, and when patients undergo transitional clinical care. The three factors that determine high-risk situations are the use of high-risk medications (such as aminoglycosides, potassium replacement or opioids), patient-related factors such as increased vulnerability at the extremes of life, and environmental factors. Based on the former three factors, the interventions in neonatal intensive care units (NICUs) should be considered as one of the highest risk situations for medication errors in the inpatient care (1). The Institute for Safe Medication Practices has reported that medication errors are more frequent in paediatric patients, and the risk of such errors causing an adverse event is higher than that in adults (2).

Some studies have estimated that one medication error occurs in every four pharmacological prescriptions issued for paediatric patients, at any instance such as prescription, distribution, preparation, or administration (3). Specifically, medication errors are common and occur at a high rate (4–6) in NICUs, and neonates in NICUs experience significantly higher medication errors and adverse drug event rates than that experienced by neonates of other wards (6). Although higher incidence of error in pediatric wards compared to neonatal wards has been also found (7), NICUs have a significantly higher rate of potential or preventable adverse drug events compared with pediatric intensive care units (6).

The introduction of electronic prescription tools in NICUs has improved the safety of the whole process of medication administration. However, it is still impossible to avoid medication errors. Pharmaceutical validation as a control measure has been shown to reduce prescription errors in other healthcare settings (6). Nevertheless, to date, the role of clinical pharmacists in NICUs has been unevenly implemented across countries, and the roles assigned to them vary. Interestingly, some studies have demonstrated that certain interventions conducted by clinical pharmacists decrease medication errors in NICU settings. However, to the best of our knowledge, specific information regarding medication error reduction after validation of electronic prescriptions generated by neonatologists for neonatal patients admitted to an NICU by a clinical pharmacist is known (8).

Given the high reported rates of medication errors in NICUs, the implementation of preventive measures to reduce such errors is imperative. In this context, the clinical pharmaceutical interventions of pharmacists make significant contributions in terms of optimised patient care and safety by rationalising prescriptions, enhancing therapeutic choices, and reducing and preventing medication errors and adverse drug effects (9–11). Therefore, this study compared, in the setting of a NICU, the outcomes of prescriptions validated by a clinical pharmacist (intervention group) and prescriptions that were not validated (control group) by determining the frequency of electronic prescription errors.

## Material and methods

### Design of the study

This study aimed to compare the frequency of electronic prescription errors when a clinical pharmacist validates a prescription vs. when pharmaceutical validation of treatments is not performed. We hypothesised that this pharmaceutical intervention can decrease the number of errors in electronic prescription and prescription error incidences in the patient.

**Figure 1:**
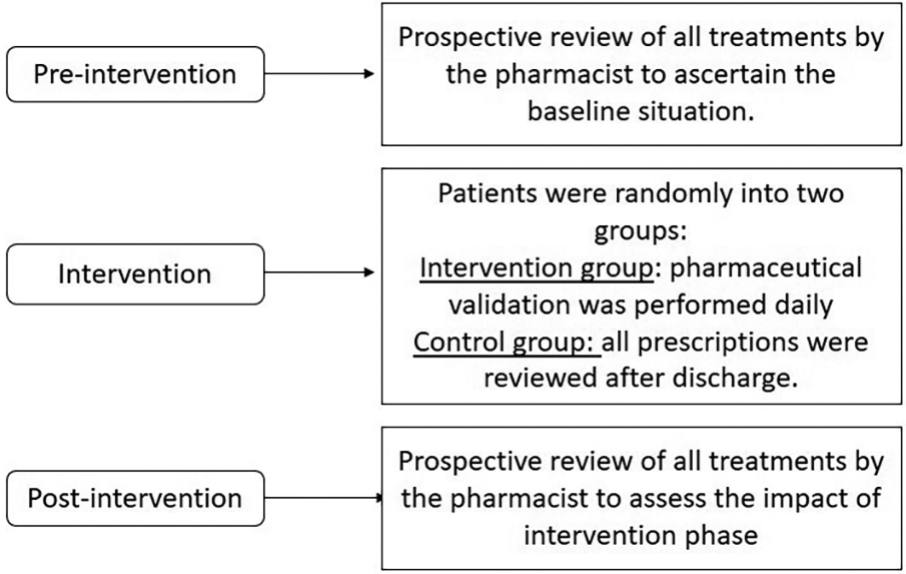
Illustration of methodology.

This was a prospective, randomised, controlled study. The pharmacist (CSMD) participated in routine newborn care during the study period and was physically present in the clinical rounds from Monday to Friday for, 2–3 h/day. This study was conducted in three phases (Figure 1):

• Pre-intervention phase: The pharmacist reviewed all prescriptions for four weeks to establish the baseline status of the neonatologists’ prescriptions.
• Randomised phase: in this phase, patients were randomly divided into control and intervention groups at admission. In the intervention group, pharmaceutical validation of prescriptions was performed daily by CSMD. In the control group, all prescriptions were reviewed after discharge from the NICU.
• Post-intervention phase: During four weeks, all neonatologists’ prescriptions were reviewed to assess the impact of the pharmacists’ presence on the daily clinical rounds in the NICU during the intervention period.

During the pre-intervention phase, the pharmacist attended daily NICU rounds and performed pharmaceutical validation of the pharmacological treatments for all NICU patients. The prescriptions were reviewed daily after the treatment was updated by the treating physicians for the patient. The prescriptions were made by the IntelliSpace Critical Care and Anaesthesia® electronic prescribing software integrated in the medical record. The elements of this e-prescription are the drug, commercial presentation, route of administration, dose, dose units, frequency, a free text field of instructions for the nursing staff, and a comment field containing specifications associated with the drug. Discrepancies encountered during the review process were communicated to the medical team responsible for the patient and resolved on the same day.

During the randomisation phase, patients were assigned to the intervention or control group at admission according to simple randomisation using a web-based system (www.dcode.fr). For patients in the intervention group, pharmaceutical validation of the treatments was performed daily in the same manner as in the pre-intervention phase. In the control group, treatments were retrospectively reviewed at discharge from the NICU by the same pharmacist. The review was conducted retrospectively because it is ethically unacceptable to detect a prescription error and not correct it or communicate it to the treating physicians. The nursing administration records were reviewed to check whether the errors found in the prescription had reached the patient.

Finally, in the post-intervention phase, all treatments were reviewed for four weeks in the same manner as in the pre-intervention phase.

### Intervention: pharmaceutical validation

Pharmaceutical validation was performed according to the paediatric pharmaceutical care model proposed by Fernández-Llamazares et al. (12). This model is based on the profile of prescription errors in the medication orders of paediatric patients admitted to eight Spanish paediatric hospitals, detected and prevented by paediatric clinical pharmacists. The model was developed using the Delphi methodology, with a panel of 50 experts. The result was a model with 39 questions based on three levels of complexity, with their respective tools for checking and perfect adaptability for other hospitals. This model includes a definition of the minimum aspects to be included in the validation process for each complexity level described, the safety profile of prescribing, types of prescribing errors identified, and the reasons that cause them, to redistribute their possible detection according to the level of complexity of the validation performed. This model distinguishes three types of validation based on the complexity level. The high complexity level of validation was selected for this study. In this type of validation, the pharmacist checks every one of the following item for each patient:

• The weight recorded in the medical record for dose calculation coincides with the birth weight until the newborn regains its birth weight. Once the newborn has regained its birth weight, the recorded weight should be updated to correspond with previous records.
• An appropriate dose per weight was not higher than the maximum dose for the indication (a deviation of 10% from the standard or protocolized dose was considered an error).
• Relationship between prescribed drugs and treatment indications for primary and secondary diagnoses.
• Appropriate pharmaceutical form and presentation.
• Facilitate access to the drug (magistral formula, foreign or controlled management drugs, or drugs not included in the hospital’s pharmacotherapeutic guide).
• Absence of interactions, duplications, and contraindications.
• Adequate dosing intervals for the patient’s corrected gestational age.
• Adequacy of the nutritional support (enteral and/or parenteral) prescribed, as well as compatibility with other prescribed drugs.
• Adjustment of drug doses in renal insufficiency cases.
• Drug levels of those drugs that are monitored in the service (e.g., aminoglycosides or antiepileptic drugs).
• Adequacy of the anti-microbial medication (the right anti-microbial, for the right indication, the right time, with the right dose, route and duration of therapy).
• Discrepancies between the prescription and the free text fields associated with it.

Prescription errors were classified according to the taxonomic criteria of the National Coordinating Council for Medication Error Reporting and Prevention and its adaptation by the Ruiz-Jarabo 2000 working group (13) into: drug not indicated/appropriate for the diagnosis; previous allergy or similar adverse effect; drug inappropriate for the patient's age or clinical situation; contraindicated drug; drug–drug interaction; drug–food interaction; therapeutic duplicity; unnecessary drug; failure to prescribe a necessary drug; higher dose; lower dose; extra dose; wrong frequency of administration; wrong route of administration; wrong rate of administration; wrong time of administration; wrong patient; longer duration of treatment; shorter duration of treatment; lack of analytical controls and discrepancy between free text and prescription. Patient-reaching errors were defined as errors detected in the prescriptions administered to patients.

### Patient inclusion criteria

All patients born during the study period who were admitted to the NICU for at least 24 h and with active pharmacological treatment were included in the study. All prescribed medications, including nutritional support, were included in this study.

Neonates with a NICU stay <24 h were excluded from the current study because, as researchers, we presumed that there was not enough time for follow-up.

### Statistical variables

The following variables were collected daily in a REDCap® (14) data collection and management tool: drugs prescribed, dose, drug dosage units, route of administration, whether the line contained any errors, the type or error and whether the error reached the patient. In addition, data were collected on gestational age, birth weight, patient characteristics, and duration of admission.

### Sample size calculation

Based on the error reduction rates published in the paediatric literature (15), a sample size of 82 patients in each group during the randomised study was calculated to estimate a significant difference in the prevalence of prescription errors ≥20%, with a 95% confidence interval and a power of 80%, assuming a 10% loss of patients. Considering an average of nine discharges per week, a duration of 18 weeks in the randomised study period was estimated.

### Statistical analyses

Population characteristics were summarised as the median (50th percentile) and interquartile range (IQR, difference between the 75th and 25th percentiles of the data), as the Kolmogorov-Smirnov hypothesis was not met for quantitative variables. Qualitative variables are expressed as absolute numbers (number of cases) and relative frequencies (percentages). The Mann–Whitney *U*-test or chi-square test was used to test for differences between the phases or between the control and intervention groups in each of the characteristics, depending on the nature of the variables. All analyses were conducted using Stata InterCooled for Windows, version 16 (StataCorp. 2019. College Station, TX, StataCorp LLC), and a significance level of 5% was assumed.

### Ethical aspects

The study was conducted in accordance with international principles and following the Patient Autonomy Act 41/2002, respecting the rights and duties established by Organic Law 3/2018 of 5 December on the Protection of Personal Data and Guarantee of Digital Rights and in accordance with Regulation (EU) 2016/679 of the Parliament and of the Council of 27 April 2016. The principles set out in the Declaration of Helsinki were adhered to, and prior to the start of the study, approval was obtained from the Center's Research Ethics Committee (Reference: 21/365) at the hospital where the study was conducted.

## Results

A total of 240 patients were included from September 2021 to June 2022: 62 patients were allocated to the pre- (*n* = 38) or post-intervention group (*n* = 24), and 178 patients were randomly divided for the randomised phase (82 newborns in the control group and 96 in the intervention group). The patient demographic characteristics are presented in Tables 1, 2.

**Table 1:** Demographic characteristics of the patients included in the pre- and post-intervention phase.

	Pre-intervention phase (*n* = 38)	Post-intervention phase (*n* = 24)	*p*-value
Sex			
Female	19 (50.0%)	11 (45.8%)	0.75
Male	19 (50.0%)	13 (54.2%)	
Median birth weight (kg)	1.96 (1.36–3)	2.42 (1.92–2.98)	0.27
Median gestational age at birth (weeks)	34 (30–38)	35 (32–38)	0.32
Patient characteristics (number and percentage)			0.165
Prematurity <32 weeks and/or low birth weight <1,500 g	14 (36.8%)	3 (12.5%)	0.036
Prematurity >32 weeks/low birth weight 1,500–2,000 g	6 (15.8%)	6 (25.0%)	0.37
Heart disease	6 (15.8%)	7 (29.2%)	0.21
Others	12 (31.6%)	8 (33.2%)	0.649
Median length of stay (days)	5.5 (3–12)	6.5 (3–13.5)	0.65
Re-admissions	0	0	–

**Table 2:** Demographic characteristics of the patients included in the randomised phase.

	Control group (*n* = 82)	Intervention group (*n* = 96)	*p*-value
Sex			
Female	49 (59.8%)	41 (42.7%)	0.74
Male	33 (40.2%)	55 (57.3%)	
Median birth weight (kg)	2.15 (1.4–3.05)	1.75 (1.09–3.16)	0.37
Median gestational age at birth (weeks)	34 (30–39)	33 (29–39)	0.49
Patient characteristics (number and percentage)			0.524
Prematurity <32 weeks and/or low birth weight <1,500 g	23 (28.0%)	37 (38.5%)	0.14
Prematurity >32 weeks/low birth weight 1,500–2,000 g	18 (22.0%)	19 (19.8%)	0.72
Heart disease	16 (19.5%)	15 (15.6%)	0.5
Others	25 (30.5%)	25 (26.0%)	0.511
Median length of stay (days)	7 (4–23)	7 (3–19)	0.35
Re-admissions	2 (2.4%)	9 (9.4%)	0.055

During the randomisation period, 5,921 prescriptions were analysed in the intervention group and 5,760 prescriptions in the control group. The median number of prescriptions per patient was 19 with an IQR of 8.0–57.5. In the intervention group, the mean was 20.5 prescriptions (IQR of 9.0–67.0) in the control group. Differences in sex, weight, gestational age at birth, or patient type between the two groups were not significant. The number of prescription errors detected was significantly lower in the intervention group than that in the control group (129 vs. 270, *p* < 0.001) because the errors in the intervention group were corrected on the day they were detected and were not perpetuated throughout the treatment. Similarly, prescription errors reaching the patient were significantly higher (40%, *n* = 108) in the control group than in the intervention group (1.6%, *n* = 2). Thus, pharmaceutical validation reduced the risk of prescription errors that reached the patient by 96%. From these results, we can state that, in the intervention group, for every three prescription errors detected, one error was prevented from reaching the patient. Table 3 describes the prescriptions of the patients included in the randomised phase, as well as the errors detected by CSMD, both the type and number of errors that reached the patient.

**Table 3:** Analysis of prescriptions in the control and intervention groups.

	Control group (*n* = 82)	Intervention group (*n* = 96)	*p*-value
Number of medical prescriptions	5,730	5,921	–
Median number of prescriptions/patient	20.5 (9–67)	19 (8–57.5)	0.66
Median number of prescriptions/patient for day	6 (5–8)	6 (5–8)	0.96
Number of prescription errors detected	270 (4.7%)	129 (2.2%)	<0.001
Number of errors/patient	1 (0–4)	0 (0–1.5)	0.058
Pharmaceutical intervention	–	125 (96.9%)	–
Type of errors			
Medication not indicated/appropriate for diagnosis	1 (0.37%)	0 (0.00%)	0.035
Previous allergy or similar adverse effect	0 (0.00%)	0 (0.00%)	
Medication inappropriate for the patient’s age or clinical situation	38 (14.07%)	20 (15.50%)	
Contraindicated drug	0 (0.00%)	0 (0.00%)	
Drug-drug interaction	0 (0.00%)	0 (0.00%)	
Drug-food interaction	0 (0.00%)	0 (0.00%)	
Therapeutic duplicity	12 (4.44%)	4 (3.10%)	
Unnecessary drug	2 (0.74%)	4 (3.10%)	
Lack of prescription of a necessary drug	18 (6.67%)	13 (10.08%)	
Higher dose	1 (0.37%)	3 (2.33%)	
Lower dose	20 (7.41%)	12 (9.30%)	
Extra dose	0 (0.00%)	0 (0.00%)	
Frequency of incorrect administration	*59* *(21.85%)*	13 (10.08%)	
Wrong route of administration	4 (1.48%)	1 (0.78%)	
Wrong rate of administration	1 (0.37%)	1 (0.78%)	
Wrong administration time	0 (0.00%)	0 (0.00%)	
Wrong patient	1 (0.37%)	1 (0.78%)	
Longer duration of treatment	1 (0.37%)	0 (0.00%)	
Lower treatment duration	0 (0.00%)	3 (2.33%)	
Lack of analytical controls	0 (0.00%)	0 (0.00%)	
Discrepancy free text—prescription	112 (41.48%)	54 (41.86%)	
Number of errors that reach the patient	108 (40%)	2 (1.6%)	<0.001

Regarding the pre- and post-intervention period, 1,240 prescriptions were analysed in the first phase and 955 in the second phase. There were no statistically significant differences between the two groups in terms of sex, weight, or gestational age at birth, although the proportion of preterm patients with a gestational age of 32 weeks at birth or weighing <1,500 g was higher in the pre-intervention group. The percentage of prescription lines with prescription errors decreased from 3.4 to 1.5% (*p* = 0.005). The reduction in the relative risk between the two periods was 56%. In both periods, the prescription of enteral nutrition resulted in the highest number of errors. No statistically significant differences were found in the type of error or medication groups. During the pre- and post-intervention periods, none of the detected prescription errors reached the patient. Table 4 shows the results of the control and intervention groups.

**Table 4:** Analysis of prescriptions in the pre- and post-intervention groups.

	Pre- intervention phase (*n* = 38)	Post-intervention phase (*n* = 24)	*p*-value
Number of medical prescriptions	1,240	955	–
Median number of prescriptions/patient	18.5 (8–46)	19 (7.5–31.5)	0.95
Median number of prescriptions/patient for day	5 (4–7)	6 (4–7)	0.84
Number of prescription errors detected	42 (3.4%)	14 (1.5%)	0.005
Number of errors/patient	0.5 (0–2)	0 (0–1)	0.25
Pharmaceutical intervention	42 (100%)	14 (100%)	–
Type of error			
Medication not indicated/appropriate for diagnosis	0 (0.00%)	0 (0.00%)	0.6
Previous allergy or similar adverse effect	0 (0.00%)	0 (0.00%)	
Medication inappropriate for the patient’s age or clinical situation	6 (14.3%)	0 (0.00%)	
Contraindicated drug	0 (0.00%)	0 (0.00%)	
Drug-drug interaction	0 (0.00%)	0 (0.00%)	
Drug-food interaction	0 (0.00%)	0 (0.00%)	
Therapeutic duplicity	1 (2.4%)	0 (0.00%)	
Unnecessary drug	1 (2.4%)	0 (0.00%)	
Lack of prescription of a necessary drug	4 (9.5%)	1 (7.1%)	
Higher dose	1 (2.4%)	1 (7.1%)	
Lower dose	2 (4.8%)	1 (7.1%)	
Extra dose	0 (0.00%)	0 (0.00%)	
Frequency of incorrect administration	5 (11.9%)	0 (0.00%)	
Wrong rate of administration	0 (0.00%)	0 (0.00%)	
Wrong route of administration	2 (4.8%)	1 (7.1%)	
Wrong administration time	0 (0.00%)	0 (0.00%)	
Wrong patient	0 (0.00%)	0 (0.00%)	
Longer duration of treatment	0 (0.00%)	0 (0.00%)	
Lower treatment duration	0 (0.00%)	0 (0.00%)	
Lack of analytical controls	0 (0.00%)	0 (0.00%)	
Discrepancy free text—prescription	20 (47.6%)	10 (71.4%)	
Number of errors that reach the patient	0	0	–

The most frequent type of error in all groups was the discrepancy between the prescriptions and free text. No significant differences were found in the distribution of prescription errors between the pre- and post-intervention groups. However, in the randomised phase of the study, there was a change in the distribution of errors in the control group, especially in the error type (frequency of incorrect dosing) because the review was performed after discharge; therefore, errors were maintained over time.

## Discussion

We conducted pre- and post-intervention studies to determine the effect of pharmaceutical validation on prescription errors in NICU. The intervention reduced prescription errors reaching the patient by 96%. In addition, in the pre-intervention period, errors occurred in 3.4% of the prescription lines, whereas in the post-intervention period, they were reduced to 1.5%. The reduction in errors may be due to both the pharmacist's presence in the unit and influence of prescription review on prescribing behaviour.

The introduction of an electronic prescription tool in our NICU has not led to the complete interception of prescription errors. Diverse studies have demonstrated that e-prescribing is a useful tool for reducing prescription errors, although its implementation has not completely eliminated them (15, 16). Studies on electronic prescribing have shown that the prescribing physician's experience can influence the frequency of prescribing errors (17). Therefore, providing adequate training in pharmacotherapy could be an effective measure in reducing such errors.

Although there are no references from Spain on the implementation of the pharmacist figure and its activities in NICUs, the role of pharmacists in these units has been described in some studies. For example, we might highlight a descriptive and exploratory study carried out by clinical pharmacists in Brazil assessed patients’ pharmacotherapy needs through visits to the neonatal unit, evaluation of prescriptions and information on medical records, identification of issues associated with pharmacotherapy, and follow-up of newborns’ clinical evolution (18). In a systematic review published in 2017, 30 studies analysed the role of pharmacists in NICU worldwide. Most studies were conducted in the United States, followed by the United Kingdom (>50%). The activities most frequently performed by pharmacists are review of patient prescriptions, prescription of parenteral nutrition, pharmacokinetic monitoring, and educational activities in the unit (8). Pharmacists’ involvement in the process of prescribing, reviewing, and preparing parenteral nutrition has been shown to reduce medication errors (19). However, no published studies have been conducted on other interventions such as pharmaceutical validation. Therefore, this is the first study to demonstrate that pharmaceutical validation reduces medication errors reaching NICU patients.

One limitation of this study is that the severity of potential medication errors was not assessed. Another limitation is that no study has been conducted on the cost-effectiveness of implementing pharmaceutical validation measures in these units. However, the daily cost of integrating a pharmacist into the unit for treatment validation is estimated to be approximately 106 euros, and it is not possible to calculate the costs avoided by preventing medication errors in our study. It is worth underlining that this was a randomised and controlled design, with a large sample of patients and a significant representation of both very preterm and very low-birth weight patients, for whom any medication error could have a relevant consequence not only in their clinical progress (18) but probably also in their neurodevelopment (20).

Given the special vulnerability of this group of patients and their susceptibility to medication errors, it is important to implement measures to reduce the number of errors that affect the patient. The pharmaceutical validation performed in this study had a positive impact on improving the quality of pharmacotherapy in NICU patients in terms of the number of errors in electronic prescribing tools and the number of prescription errors reaching the patient, compared to the absence of intervention.

The high technological complexity of neonatal services requires updated knowledge, protocolization, and assessment of the work. In the face of medication errors, the pharmacist is in the right position to analyse and correct, improve communication between professionals, and promote best practices. Clinical pharmacy interventions in NICUs can benefit safety that focuses on preventing medication errors. In particular, our results prove that clinical pharmacists play an essential role in medical treatment by identifying and intercepting prescription errors; therefore, they should be made an integral part of the NICU staff. This requirement is in line with the objectives of the World Health Organization in its third global challenge of patient safety.

## Data Availability

The raw data supporting the conclusions of this article will be made available by the authors, without undue reservation.
